# Adverse Childhood Experiences Are Linked to Age of Onset and Reading Recognition in Multiple Sclerosis

**DOI:** 10.3389/fneur.2017.00242

**Published:** 2017-06-02

**Authors:** Michael T. Shaw, Natalie O. Pawlak, Ariana Frontario, Kathleen Sherman, Lauren B. Krupp, Leigh E. Charvet

**Affiliations:** ^1^Department of Neurology, NYU Langone Medical Center, New York, NY, United States; ^2^Lake Erie College of Osteopathic Medicine, Erie, PA, United States

**Keywords:** adverse childhood experiences, multiple sclerosis, age of onset, premorbid IQ, childhood abuse

## Abstract

**Background:**

Adverse childhood experiences (ACEs) exert a psychological and physiological toll that increases risk of chronic conditions, poorer social functioning, and cognitive impairment in adulthood.

**Objective:**

To investigate the relationship between childhood adversity and clinical disease features in multiple sclerosis (MS).

**Methods:**

Sixty-seven participants with MS completed the ACE assessment and neuropsychological assessments as part of a larger clinical trial of cognitive remediation.

**Results:**

Adverse childhood experience scores, a measure of exposure to adverse events in childhood, significantly predicted age of MS onset (*r* = –0.30, *p* = 0.04). ACEs were also linked to reading recognition (a proxy for premorbid IQ) (*r* = –0.25, *p* = 0.04). ACE scores were not related to age, current disability, or current level of cognitive impairment measured by the Symbol Digit Modalities Test (SDMT).

**Conclusion:**

Childhood adversity may increase the likelihood of earlier age of onset and poorer estimated premorbid IQ in MS.

## Introduction

Childhood adversity and the associated chronic psychological and physiological stress have been shown to lead to poorer health in adults, including susceptibility to proinflammatory states and chronic conditions (1–3). Adults with a history of traumatic experiences in childhood have been found to have smaller prefrontal cortex (4, 5) and hippocampal volume (6) and greater activation of the hypothalamic–pituitary–adrenal (HPA) axis (7) (which mediates the body’s physiological response to psychosocial and environmental stress) compared to non-maltreated individuals. Moreover, previous studies have linked history of childhood adversity to biomarkers of inflammation, such as C-reactive protein, that increase susceptibility to autoimmune diseases in adulthood (1, 2, 8–10).

Multiple sclerosis (MS) is a complex autoimmune disorder that destroys the myelin sheath and neurons in the brain and spinal cord, resulting in lesions, plaques, and scars at the inflamed sites. Recent studies have made progress in identifying the genetic and environmental components of disease vulnerability (e.g., exposure to tobacco smoke, Epstein–Barr virus (EBV) infection, and inadequate vitamin D levels), but the exact etiology of MS is still unknown. In patients with MS, hyperactivity of the HPA axis was found to be correlated with MRI activity, clinical disability, and cognitive impairment (11–13). Exposure to major negative life events was found to be predictive of gadolinium-enhancing and T_2_ lesions on MRI scans in patients with MS (14). Studies looking at psychosocial factors influencing disease onset found that cohorts of participants with MS had a higher prevalence of negative life events, self-defined family problems, and poorer utilization of social support resources compared to healthy controls (15).

Adverse events occurring as early as childhood have been linked to MS clinical disease features. For example, emotional and physical abuse and neglect in childhood have been associated with increased rates of relapses in a cohort of adult MS patients (16). However, the exact role of early-life adversity in MS remains unclear. Determining the physiologic effects of childhood stress in the context of psychophysiological vulnerability to MS onset and progression is important for implementing more effective and early approaches to improve resilience.

The adverse childhood experience (ACE) inventory is a validated, reliable, and easily implemented assessment of childhood adversity through a study including 17,337 adult participants conducted by the CDC and the Kaiser Foundation Health Plan in San Diego, California (17). In this study, ACE scores were found to be highly predictive of risk for a number of chronic conditions and emotional dysfunction in adulthood. An ACE score of 4 or more exposures denoted a severely adverse childhood that exponentially increases risk of chronic conditions, suicide attempts, psychiatric disorders, poor self-rated health, physical inactivity, obesity, ≥50 sexual intercourse partners, sexually transmitted diseases, and harmful lifestyle choices such as smoking compared to those who had not experienced any adverse childhood events (3). In this study, we tested the relationship between childhood adversity and MS clinical disease features using the ACE survey.

## Materials and Methods

### Study Selection

The study participants were individuals with MS recruited from a larger sample of *n* = 135 participants with MS who had completed a 12-week clinical trial of a cognitive remediation program at Stony Brook Medicine (18). Following study completion, participants were contacted by phone with the opportunity to participate in an online survey study from home.

Written, informed consent was obtained from all participants before the initiation of any specific procedures. Study procedures were approved from Stony Brook University’s Human Subjects Committee Institutional Review Board and the CORIHS B, board reference number: 2013-2110-R3. Participants were actively recruited for the cognitive remediation trial between July 2014 and June 2015.

The following were included as eligibility criteria for the larger trial: age between 18 and 70 years; definite diagnosis of MS (of any subtype) by a treating neurologist; lack of other primary CNS or medical disorder that would influence ability to participate; lack of other uncontrolled serious medical conditions (e.g., cancer); no history of alcohol or other substance abuse disorder; native proficiency of the English language (defined as learning English before the age of 12 years). As part of the larger cognitive remediation trial, participants were also required to have at least suspected cognitive impairment, as measured by scoring at least 1 SD or below on the Symbol Digit Modalities Test (SDMT) (19) and within the average range on Wide Range Achievement Test-third edition (WRAT-3) (20).

### Cognitive Measures

For the current study, the SDMT screening and WRAT-3 reading subtest scores from the clinical trial were used to represent current cognitive information processing and oral reading recognition, respectively. The SDMT is a brief information processing measure that is widely accepted measure of MS-related cognitive involvement (21). Reading recognition as measured by the WRAT-3 (22) is a commonly accepted proxy of estimated premorbid IQ (23–25). These measures were administered from 4 to 18 months prior to the completion of the survey; however, both measures have well-demonstrated reliability over time and would not be expected to change significantly over this time period (26).

### Childhood Adversity Questionnaire

The ACE questionnaire is a 10-item measure of childhood adversity including household dysfunction, neglect, and emotional, physical, and sexual abuse. Exposure to each adverse event counts as a point toward one’s ACE score, with higher ACE scores indicating greater exposure to traumatic and stressful events in childhood (3). Of note, an ACE score of 4 or more exposures represents a severely adverse childhood that exponentially increases risk of chronic conditions, suicidality, psychiatric disorders, obesity, and other health outcomes in adulthood compared to those without ACEs. In this study, we correlated ACE scores with clinical disease features in our cohort of MS patients using IBM SPSS Statistics 23. Baseline characteristics for the ACE questionnaire are shown in Table 1.

**Table 1:** Childhood adversity descriptors—primary measures.

Measure	Sample size	Mean score (SD)
Adverse childhood experience (ACE) score	*n* = 67	1.93 (2.45)
ACE *z*-score	*n* = 67	0.053 (0.94)
ACE (range)	*n* = 67	0.00–10.00

## Results

Of the *n* = 135 cognitive remediation trial participants, *n* = 79 responded with interest in this study and *n* = 67 fully completed the online ACE survey. Demographic and clinical features for the *n* = 67 participants are shown in Table 2.

**Table 2:** Demographic and baseline clinical characteristics.^a^

Demographic characteristics		
Female gender, *n* (%)	*n* = 67	77.60%
Age (years), mean (SD)	*n* = 67	50.49 (10.67)
Age range (years)	*n* = 67	22–69
**Clinical characteristics**		
EDSS, median (range)	*n* = 59	4.00 (0.00–8.00)
Age of onset (years), mean (SD)	*n* = 46	32.40 (11.67)
Disease duration (years), mean (SD)	*n* = 46	16.92 (10.26)
Education (years), mean (SD)	*n* = 67	15.28 (2.54)
Multiple sclerosis (MS) subset, *n* (%) Relapse-remitting MS Secondary progressive MS Primary progressive MS Unknown (indistinguishable between relapse-remitting and progressive subtype)	*n* = 67	*n* = 42 (62.70%)*n* = 17 (25.40%)*n* = 6 (9.0%)*n* = 2 (3.0%)

*^a^Characteristics provided in table are based off of most accurate and available data in study for n = 67 participants*.

**Figure 1:**
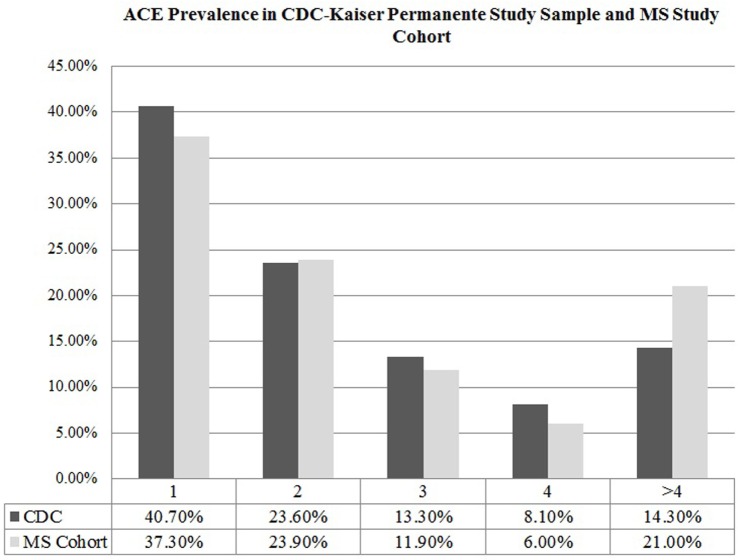
Adverse childhood experience prevalence in CDC-Kaiser Permanente study sample and multiple sclerosis (MS) study cohort.

The mean ACE score of our cohort was 1.93 ± 2.45, denoting that on average, participants experienced two ACEs in their lifetime. Comparing the ACE distribution of our study cohort to the prevalence found in the CDC-Kaiser Permanente ACE study sample, our cohort of MS patients reported a higher number of ACEs (Figure 1), with more individuals in our MS sample meeting criteria for a severely adverse childhood that exponentially increases health risk in adulthood compared to those in the CDC-Kaiser Permanente cohort.

**Figure 2:**
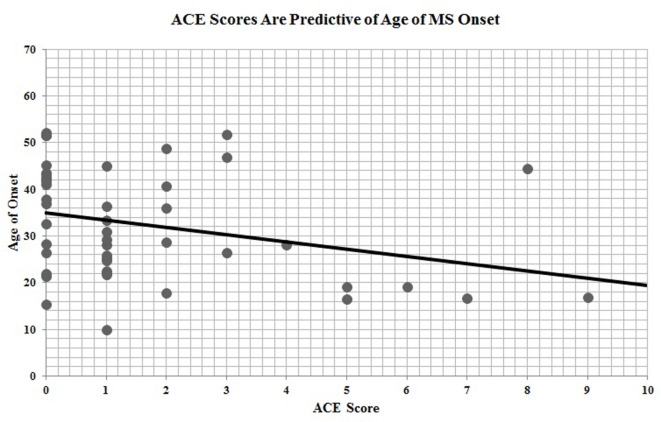
Adverse childhood experience (ACE) scores are predictive of age of multiple sclerosis onset.

Of the 67 participants with ACE data, *n* = 46 had provided date of disease onset. ACE scores were significantly and inversely correlated with age of MS onset (*r* = −0.30, *p* = 0.04), indicating that a greater exposure to ACEs is predictive of earlier age of disease onset (Figure 2).

**Figure 3:**
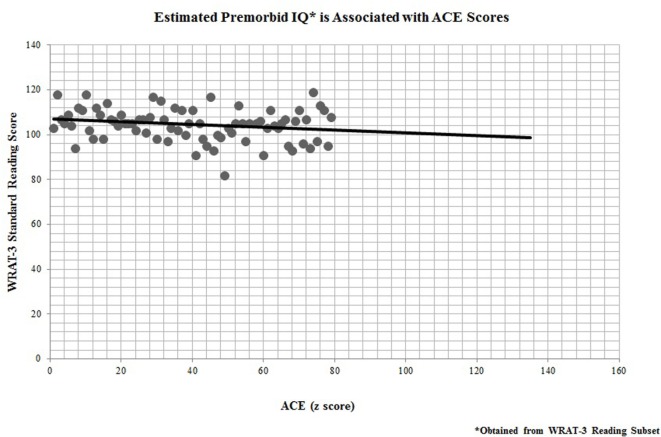
Estimated premorbid IQ* is associated with adverse childhood experience (ACE) scores. *Obtained from Wide Range Achievement Test-third edition (WRAT-3) Reading Subset.

In addition, all 67 participants who completed the ACE questionnaire also performed neuropsychological testing for cognitive remediation measures. Performance on the WRAT-3 reading recognition was significantly linked to participants’ ACE scores (*r* = −0.25, *p* = 0.04; Figure 3), indicating that childhood adversity was also predictive of premorbid IQ.

## Discussion

In this survey study, adults with MS reported ACEs that were consistent with those found in the larger epidemiologic study conducted at Kaiser Permanente, with the exception of an overrepresentation of participants meeting criteria for a severely adverse childhood (i.e., 4 or more ACEs). Further, when assessing ACE scores, those participants reporting greater childhood adversity had significantly earlier age of disease onset. ACE scores were also predictive of estimated premorbid IQ, but were not predictive of SDMT scores, which serve as a measure of current MS-related cognitive involvement.

The study results suggest that physiological vulnerability to MS disease is linked to childhood exposure to adverse experiences. There are several considerations in linking childhood adversity to MS onset. These findings are consistent with previous literature suggesting that childhood adversity induces physiological dysregulation that increases the risk of developing chronic conditions as an adult (1–3, 27). Uncovering the link between childhood adversities and disease onset helps to build our understanding of the cumulative effects of stress on long-term health and MS susceptibility.

This study has several major limitations. First, there are additional potentially mediating factors to the findings that were not available such as socioeconomic status and nutrition in childhood that would likely correspond to higher ACE scores or mitigating factors such as social support that could reduce the impact of childhood adversity (28). In addition, there are numerous known and suspected factors that contribute to the onset of MS [e.g., geographic location (29), vitamin D levels (30), and additional genetic and environmental factors (31)] that would add to a richer interpretation of the current findings.

One of the possible mechanisms through which childhood adversity influences physiological dysregulation is changes in biological processes known as allostatic mechanisms. Through allostatic mechanisms, the body mobilizes adaptive processes in response to environmental and psychosocial stressors (32). In the short term, these processes are protective. However, chronic and repeated exposure to extreme stress can lead to a physiological “wear and tear” of the body, either through multiple cycles of allostasis or through the inefficient turning on and shutting off of allostatic responses (32). The overtaxing of adaptive processes mediated by the endocrine, immune, and nervous systems in allostasis is defined as allostatic load, which leads to harmful physiological dysregulation. For example, allostatic load involves glucocorticoid hyperactivity induced by prolonged and repeated exposure to stress that has been associated with reduced memory performance, most likely due to decreased neurogenesis and neuroplasticity in glucocorticoid-sensitive brain areas such as the hippocampus (33–35). Future studies in MS should investigate biometric indices to mark the presence of stress-induced physiological overtaxing across the lifespan and to better understand the role of physiologic stress relative to MS vulnerability and cognitive involvement. Various validated measures of allostatic load exist that could be implemented in clinical visits to assess stress-induced wear and tear of allostatic mechanisms across the lifespan of MS patients. These measures of allostatic load could be compared to participants’ reported ACE scores to hone in on how childhood adversity influences physiological “wear and tear” that promotes biological vulnerability to MS and early-age onset.

A few factors are important to consider in the generalizability of our findings. First, through surveying those participants in our clinical trial of cognitive remediation, we included a sample with probable cognitive impairment. While this is a common feature of MS, with impairment on the SDMT is estimated at up to 70% of MS in general (36), this sample did not represent those patients living without cognitive involvement of the disease. Also, by surveying trial participants up to 18 months after study completion, the timing of the cognitive measures was not concurrent with the survey data in some cases. However, both of the cognitive measures are expected to be generally stable over time and would not be expected to significantly influence our findings (20, 26). Our study cohort had a gender distribution that was predominantly female (77.60%), unlike the sample in the original ACE study conducted by the CDC and Kaiser Permanente, which had a relatively even gender distribution (54% female). Research has shown that women are more likely than men to experience sexual abuse and neglect in childhood (37, 38). There are also proven sex differences in severity of reaction to traumatic experiences, with women being more prone to develop PTSD and hence, stress-induced physiologic dysregulation (38). Both of these factors could contribute to the greater prevalence of self-reported severely traumatic childhoods (defined as having an ACE score equal to or greater than 4) in our MS study sample compared to the ACE study sample. Nevertheless, these gender differences have also been observed in MS and other autoimmune disorders, with women being two to three times more likely to develop MS compared to male counterparts (39). This study’s participants exhibited MS-related cognitive impairment and provided an additional limitation to obtaining self-reported measures on childhood experiences for the full range of the MS population.

## Conclusion

This is the first study, to our knowledge, to examine the influence of ACE scores in MS clinical features. Findings support a role of childhood adversity in MS disease onset. Ultimately, therapeutic interventions for stress management may be useful, as well as interventions in childhood that focus on the development of strategies of resilience. Childhood exposure to psychosocial stressors, early trauma-induced reactions, and resilient coping strategies for stress management should be considered along with other predictive factors to promote healthier aging and to prevent cognitive decline across the lifespan in MS patients.

## Ethics Statement

The study protocol was approved by and was carried out in accordance with the recommendations of the Stony Brook University’s Human Subjects Committee Institutional Review Board (IRB) and the CORIHS B, board reference number: 2013-2110-R3, with written or verbal informed consent obtained from all subjects. All subjects gave written or verbal informed consent in accordance with the Declaration of Helsinki.

## Author Contributions

All mentioned authors have significantly contributed to the manuscript as outlined by the Frontiers Multiple Sclerosis and Neuroimmunology authorship guidelines. All authors participated in drafting and revising the work and in the final approval process, and all agree to be accountable for all aspects of the work in ensuring that questions related to the accuracy or integrity of any part of the work are appropriately investigated. Corresponding author LC and author KS made substantial contributions to the conception and design of the work; authors MS, NP, and AF significantly contributed to the acquisition, analysis, and interpretation of collected data.

## Conflict of Interest Statement

The authors declare that the research was conducted in the absence of any commercial or financial relationships that could be construed as a potential conflict of interest.
